# A functional comparison of the domestic cat bitter receptors Tas2r38 and Tas2r43 with their human orthologs

**DOI:** 10.1186/s12868-015-0170-6

**Published:** 2015-06-03

**Authors:** Michelle M Sandau, Jason R Goodman, Anu Thomas, Joseph B Rucker, Nancy E Rawson

**Affiliations:** AFB International, St. Charles, MO USA; Integral Molecular, Inc., Philadelphia, PA USA

**Keywords:** Bitter, flavor, feline, domestic cat, taste, Tas2r38, Tas2r43

## Abstract

**Background:**

Domestic cats (*felis catus*) have a reputation for being rather unpredictable in their dietary choices. While their appetite for protein or savory flavors is consistent with their nutritional needs, their preference among protein-sufficient dietary options may relate to differences in the response to other flavor characteristics. Studies of domestic cat taste perception are limited, in part, due to the lack of receptor sequence information. Several studies have described the phylogenetic relationship of specific cat taste receptor sequences as compared with other carnivores. For example, domestic cats are obligate carnivores and their receptor Tas1r2, associated with the human perception of sweet, is present only as a pseudogene. Similarly, the cat perception of bitter may differ from that of other mammals due to variations in their repertoire of bitter receptor (*Tas2r*) genes. This report includes the first functional characterization of domestic cat taste receptors.

**Results:**

We functionally expressed two uncharacterized domestic sequences Tas2r38 and Tas2r43 and deorphanized the receptors using a cellular functional assay. Statistical significance was determined using an unpaired, two-tailed t-test. The cat sequence for Tas2r38 contains 3 major amino acid residues known to confer the taster phenotype (PAI), which is associated with sensitivity to the bitter compounds PROP and PTC. However, in contrast to human TAS2R38, cat Tas2r38 is activated by PTC but not by PROP. Furthermore, like its human counterpart, cat Tas2r43 is activated by aloin and denatonium, but differs from the human TAS2R43 by insensitivity to saccharin. The responses of both cat receptors to the bitter ligands were concentration-dependent and were inhibited by the human bitter blocker probenecid.

**Conclusions:**

These data demonstrate that the response profiles of the cat bitter receptors Tas2r38 and Tas2r43 are distinct from those of their orthologous human receptors. Results with cat Tas2r38 also demonstrate that additional residues beyond those classically associated with PROP sensitivity in humans influence the sensitivity to PROP and PTC. Functional studies of the human bitter receptor family are being applied to the development of food and medicinal products with more appealing flavor profiles. Our work lays the foundation for similar work applied to felines.

**Electronic supplementary material:**

The online version of this article (doi:10.1186/s12868-015-0170-6) contains supplementary material, which is available to authorized users.

## Background

Taste is a key mechanism used by animals for the selection of foods that are nutritive and safe for ingestion, and it plays an important role in influencing animal health and disease. For example, umami and sweet taste are used to select for food that are rich in calories and amino acids while bitter taste is typically used to avoid food containing potential toxins [1]. The perception of bitter taste is mediated by TAS2Rs, a family of G protein-coupled receptors (GPCRs). TAS2Rs are expressed on the apical surface of bitter taste cells and transduce intracellular signals through the activation of intracellular heterotrimeric G proteins [2-4]. Currently, it is believed that a subset of human TAS2Rs is promiscuous, activated by multiple ligands across chemical classes, while other human TAS2Rs bind ligands of only particular chemical classes [5]. Furthermore, multiple human TAS2Rs are orphan receptors, with no compounds known to stimulate them [6]. Collectively, the human repertoire consists of at least 25 TAS2R sequences, which contain more than 80 single nucleotide polymorphisms (SNPs) [7,8]. These receptors recognize bitter compounds that are diverse in chemical structure and are found in plants [9] or are created through fermentation and Maillard reactions [10, 11]. Compounds that block human bitter taste have also been identified [12-14]. It is the goal of many pharmaceutical and food manufacturers to identify compounds that either block or alter bitter perception, thus creating a more palatable product.

The ability of mammals to taste the five primary modalities is generally thought to be largely similar [15, 16], yet perceptual differences exist between individuals and among species. One explanation for variations between individuals is amino acid differences within a given receptor, which can potentially alter the repertoire of ligands that bind and the intracellular signals generated once stimulated [17-20]. Furthermore, diet and environmental differences have resulted in taste receptor evolution across mammalian species wherein different specificities and sensitivities exist [21-23]. More dramatic perceptual differences may be explained by the loss of functional receptors. Notably, the Tas1r2 protein, a component of the receptor for sweet compounds, has mutated to a nonfunctional pseudogene in felines [24]. A similar loss of taste genes is observed among several other obligate carnivores, including aquatic mammals such as dolphins, which have undergone a drastic loss to the point where they lack most functional taste receptor genes [25]. Accordingly, generalizations across species regarding taste perception may be unreliable.

Studies of bitter taste in felines are limited. Early neurophysiological work on domestic cats recorded responses to orally delivered bitter stimuli from the chorda tympani and geniculate ganglion [26, 27]. These studies reported responses to quinine, including both inhibition and activation, that were spread across multiple classes of neurons, and that in some cases were adapted by pre-exposure to acidic stimuli, suggesting a lack of specificity. Behaviorally, cats reject quinine and are reported to be more sensitive to quinine than humans [26,28-31], and denatonium sulfate has been used in commercial products to deter cats from chewing on furniture and other objects.

Obligate carnivores consume little to no plant material, a primary source of potentially toxic bitter compounds; therefore, the utility of bitter receptors in these animals is not readily apparent. However, plant constituents are found in the stomachs of feral domestic cats, wild cats and their hybrids [32, 33]. This plant material may be through direct consumption or through indirect intake of the gastrointestinal tract of prey animals and additional bitter compounds may be present in certain tissues and bile. Furthermore, the composition of domestic cat food includes grains and umami flavors generated from Maillard reactions, which can lead to the generation of bitter compounds [10]. Bitter taste receptors can also detect bacterial metabolites, such as quorum sensing compounds, and bitter taste may be involved in detection of rotten food [34-36]. Thus domestic cats have the opportunity to encounter bitter compounds which could influence their preference in either their offered food or while foraging.

The availability of genetic data has vastly facilitated our understanding of bitter taste perception in many species through functional expression of taste receptors [3, 5, 13, 19, 37-43]. These include human TAS2R38, which responds to the thiourea-containing molecules phenylthiocarbamide (PTC) and 6-propyl-2-thiouracil (PROP) [19]; human TAS2R43, which responds to saccharin and aloin [20, 41, 44]; and human TAS2R16, which responds to β–glucosides such as salicin [38]. Bitter taste receptors, including these, in general show a striking correspondence between the functional activity of different allelic variants as measured in cells and perceptual specificity and sensitivity. For example, psychophysical data of individuals with the TAS2R38 PAV ‘taster’ genotype of TAS2R38 correlated with low *in vitro* EC_50_ values for PTC and PROP while the AVI ‘nontaster’ phenotype correlated with high *in vitro* EC_50_ values [19]. Similarly the activation profiles of human TAS2R43 and TAS2R31 [41] and TAS2R16 [45] allelic variants correlated with the psychophysical data determined for the bitter compounds in human studies. Due to the close correlation of human psychophysical data and *in vitro* data we focused this study on the initial deorphanization of two domestic cat bitter taste receptors using cellular assays. Future studies will focus on the deorphanization and characterization of the remaining receptors and will aid in the interpretation of historical data and design of future behavioral studies with cats.

Since the sequencing of the domestic cat genome [46, 47], no published studies have examined cat taste perception through functional expression of cat taste receptors. The extent of the carnivore bitter taste receptor repertoire analysis to date has focused primarily on phylogenetic relationships among sequences and repertoire size [21, 48, 49]. Humans and mice encode a moderate number of receptors at 25 and 34 respectively. Species at the extreme of this spectrum are the frog, which encodes about 50 receptors while the chicken encodes only 3 [50, 51]. Public databases such as NCBI predict 13 domestic cat genes encoding bitter taste receptors, and Ensembl predicts at least 7 such genes. Our studies began prior to these annotations and we identified sequences through a BLAST query against the domestic cat genome. We chose to pursue two gene sequences predicted to encode TAS2R38 and TAS2R43 equivalents on the basis of their sequence similarity to these human receptors. The ortholog to TAS2R38 was chosen due to high sequence similarity, while the TAS2R43 ortholog is similar to a family of human receptors that have a broad range of specificities.

In this study we identified, functionally expressed, and deorphanized two cat genes predicted to encode orthologs of the human bitter taste receptors TAS2R38 and TAS2R43. On the basis of specific amino acid conservation in the domestic cat sequences we hypothesized the receptors had a reasonable likelihood to respond to the human bitter compounds activating their human orthologs. Our data indicate a response profile by the cat bitter receptors that are distinct from that of their human counterparts. We additionally report an unexpected Tas2Rr38 response profile to PTC and PROP.

## Results and Discussion

To understand the cellular and molecular determinants of cat taste perception we began by identifying and cloning cat genes predicted to encode proteins corresponding to two human bitter taste receptors, TAS2R38 and TAS2R43. The human TAS2R38 and putative cat Tas2r38 protein sequences are 67.6% identical (Additional file 1: Figure S1). The three most common human TAS2R38 polymorphisms which are associated with taste sensitivity to PTC and PROP occur at amino acid position 49, where either a proline or an alanine is encoded; at position 262, where either an alanine or valine is encoded; and at position 296, where either a valine or an isoleucine is encoded. These polymorphisms result in two frequent human haplotypes PAV and AVI, associated with the taster and non-taster phenotypes, respectively [19, 52]. At the equivalent amino acid positions in the cat protein, the sequence displays an apparent intermediate taster genotype of PAI. A human TAS2R38 engineered with this haplotype responded nearly equivalently to the PAV taster haplotype when stimulated with PROP and PTC in cellular assays [19]. Given these similarities we hypothesized that the cat ortholog of human TAS2R38 would respond to the human ligands PTC and PROP.

**Fig. 1:**
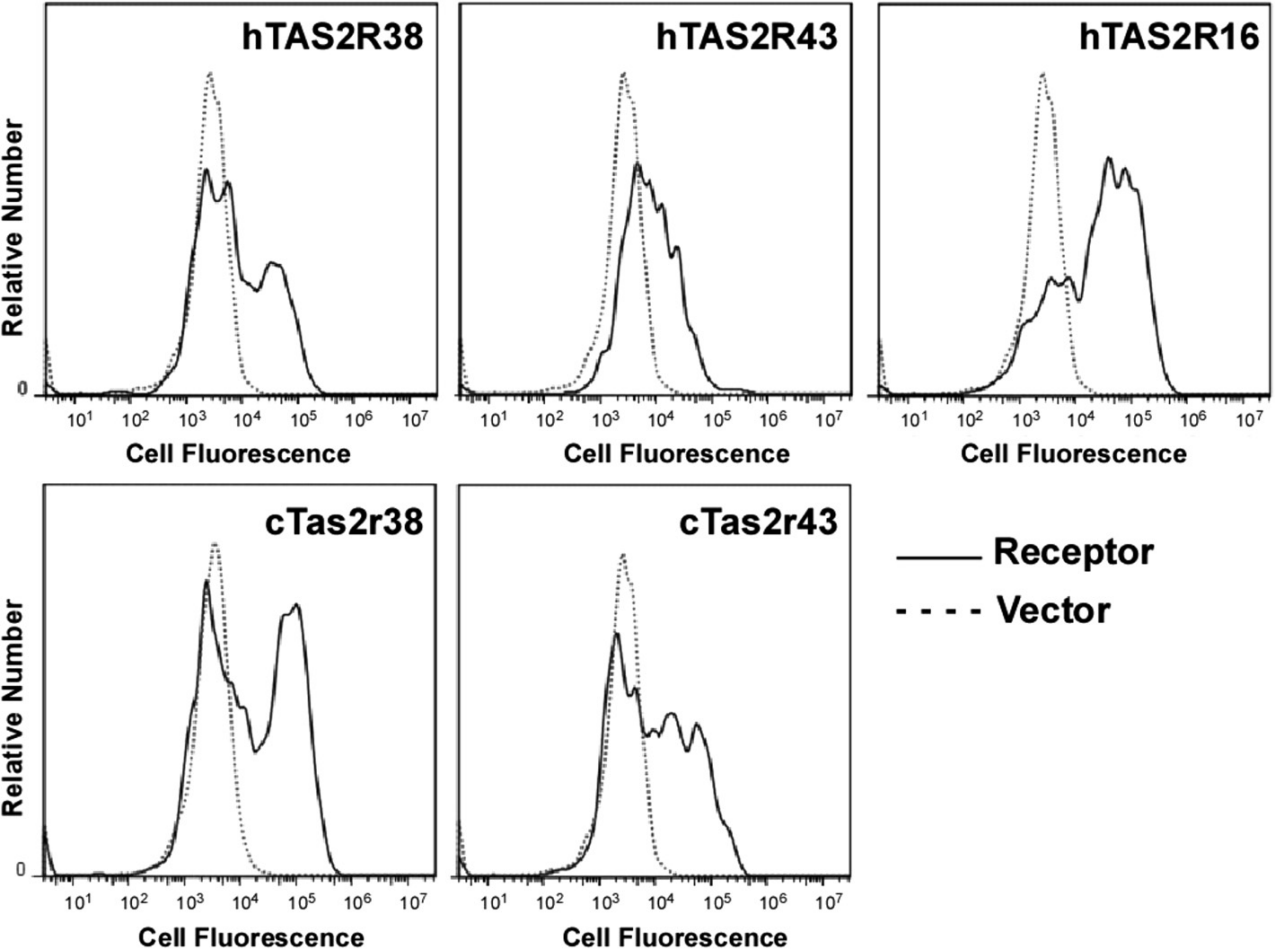
Cat Tas2r38 and Tas2r43 express on the cell surface similarly to their human orthologs. Each of the expression constructs was transfected individually along with Gα16-gust44. Expression of the receptors on the cell surface was determined 24 hours after transfection by flow cytometry of fixed cells, using a mAb recognizing the FLAG sequence encoded at the receptor N-terminus, which is exposed at the extracellular surface of TAS2R receptors. Shown are the fluorescence profiles for cells transfected with specific human (h) or cat (c) TAS2R expression constructs (black), or with vector alone (dotted line). Human TAS2R16 is included as a control as it consistently and robustly traffics to the cell surface.

We also identified in the domestic cat genome a TAS2R sequence that clusters with the TAS2R43-like family. Human TAS2R43 belongs to a subfamily of receptors including human TAS2R30, 31, 45 and 46 [53]. The cat genome also contains an additional bitter receptor with sequence similarities to this receptor family, but was not pursued in these studies due to low expression levels in our cellular assay. Within the Ensembl database, Felis catus 6.2 build Gene: ENSFCAG00000030153 is 99.3% similar to our sequence. We chose to identify this receptor as cat Tas2r43 due to the response profile to the ligands in the experiments described below. Cat Tas2r43 encodes a protein that is 59% identical to the human TAS2R43 receptor (Additional file 1: Figure S1B). In human TAS2R43, a tryptophan in position 35 is an allele that makes humans sensitive to the bitterness of aloin [20, 54]. This tryptophan is conserved in the cat sequence, thus we hypothesized that the cat receptor may respond similarly to the aloin-sensitive human receptor despite its modest overall sequence similarity.

Cellular experiments were conducted to deorphanize these two cat bitter receptors. To monitor cat and human bitter receptor activation and inhibition we used an *in vitro* calcium flux assay with receptors transiently expressed in a mammalian cell line that does not endogenously express bitter receptors or respond to the selected ligands [12]. The human and cat bitter genes were expressed with an encoded N-terminal epitope sequence allowing for detection of cell surface-expressed receptor, followed by a SST3 export sequence that allows efficient bitter receptor trafficking to the cell surface [12]. We chose six known agonists of human TAS2R38 or TAS2R43 as candidate ligands to the cat receptors (Additional file 2: Figure S2).

Transient transfection of either of the putative cat receptors resulted in expression of the protein on the surface of the cell, as detected by flow cytometry using the N-terminal epitope sequence (Fig. 1). A control receptor, human TAS2R16, was expressed on the surface of 36% of the cells. This receptor was included as it consistently and robustly traffics to the cell surface and responds to its agonist, salicin, in cellular assays [12, 38]. In addition, human TAS2R16 does not respond to any known ligands of human TAS2R38 and TAS2R43 [5]. Human TASR38 and cat Tas2r38 were expressed in a similar percentage of cells (in 32% and 36% of cells, respectively), with the expression level of the cat receptor having a slightly higher mean fluorescence intensity than the human equivalent (16.1 × 10^4^ and 8.6 × 10^4^ relative fluorescence units (RFU), respectively). The expression of cat Tas2r43 and human TAS2R43 was also similar (in 34% and 35% of cells, respectively), with a similar level of expression (mean fluorescence intensity 3.8 × 10^4^ and 2.8 × 10^4^ RFU, respectively). The cat receptors trafficked to the surface of the cell at levels comparable to the human TAS2R16 control, allowing us to experimentally investigate their responses to agonists.

### Deorphanization of cat Tas2r receptors

In cells expressing the transiently transfected bitter receptor and chimeric G protein, addition of a cognate agonist induces an increase in intracellular calcium levels that can be measured using a Ca^2+^-activated fluorescent dye. We performed such experiments on the predicted cat Tas2r38 and Tas2r43 receptors, with the equivalent human receptor used as an internal control for potential ligands. The human TAS2R38 and TAS2R43 responses to these compounds have been reported [5, 19, 20, 38, 41, 44], while the human TAS2R16 receptor served as a negative control to demonstrate the specificity of the assay.

**Fig. 2:**
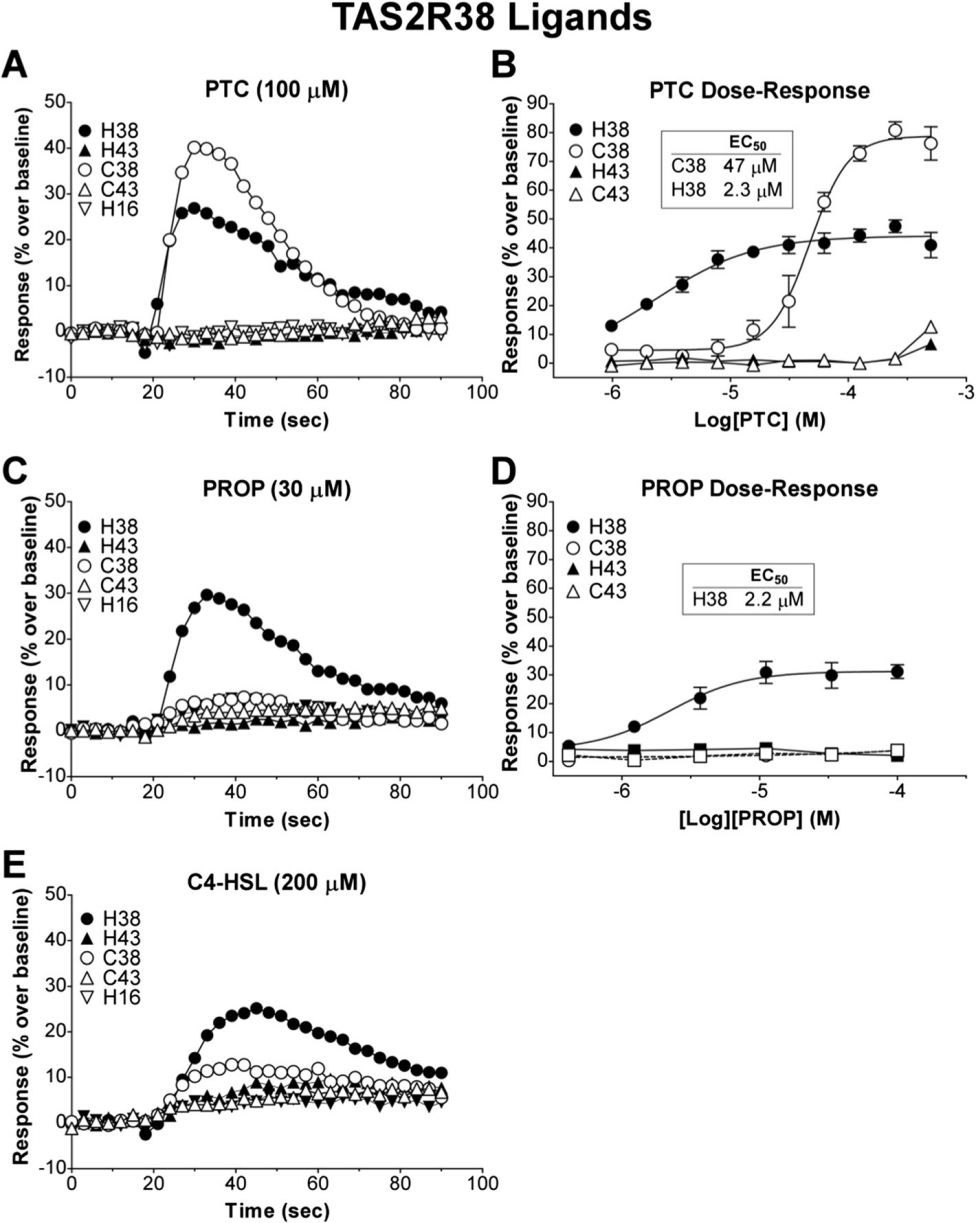
Human and cat TAS2R38 show different ligand responses and affinities. Cells were transiently transfected with Gα16-gust44 and the indicated receptors human TAS2R38 (H38) TAS2R43 (H43) and TAS2R16 (H16), and cat Tas2r38 (C38) and Tas2r43 (C43). 22 hours post-transfection, calcium influx was measured in cells challenged with the ligands characteristic for human TAS2R38. (**a **& **b**) Both human and cat TAS2R38 responded to PTC in a dose-dependent manner (individual trace shown for 100 μM); however, the dose response shows the cat Tas2r38 EC_50_ (47 μM) was at least 10-fold lower than that of human TAS2R38 (2.3 μM). (**c **& **d**) Only human TAS2R38 responded to PROP. (**e**) Both human and cat TAS2R38 responded to C4-HSL

In cells expressing either the human TAS2R38 or the cat ortholog receptor, PTC at 100 μM resulted in a strong calcium flux (Fig. 2a). The magnitude of the response by the cat receptor was higher than the human receptor (peak fluorescence of 40 % vs. 27 % over baseline; Fig. 2a). A dose–response analysis of the human and cat receptors with PTC results in an EC_50_ of 47 μM for the cat Tas2r38 while the EC_50_ for human TAS2R38 is only 2.3 μM (Fig. 2b). Thus the cat receptor is at least 10-fold less concentration sensitive to PTC than the human receptor (albeit with a stronger cellular response). The different EC_50_ and maximal response could be explained by the increased surface expression of the cat receptor or distinct G protein coupling and the intracellular signals generated. Human TAS2R43, TAS2R16 and predicted cat Tas2r43 did not respond to PTC, demonstrating the specificity of the response.

Cells transfected with the cat Tas2r38 did not respond to PROP at 30 μM, in contrast to cells expressing human TAS2R38 (Fig. 2c). Dose–response studies of PROP with the cat receptor show no response to PROP at concentrations 50-fold greater than the EC_50_ for PROP with the human receptor (Fig. 2d).

Lastly, we tested the known human TAS2R38 agonist compound C4-HSL (Fig. 2e), a small molecule secreted by bacteria as a quorum sensor [35]. The human and cat TAS2R38 receptors responded weakly but specifically to C4-HSL while TAS2R43 and TAS2R16 demonstrated no response. The EC_50_ for C4-HSL was not calculated for either species’ TAS2R38 receptor due to an increase in non-specific calcium influx seen at higher concentrations potentially due to cell poration or lysis. However, at 200 μM a clear and statistically significant calcium flux was observed when TAS2R38 was compared with TAS2R43 (Additional file 3: Figure S3A). Similarly, the calcium flux in response to C4-HSL by cat Tas2r38 was significantly different than cat Tas2r43 (Additional file 3: Figure S3B).

**Fig. 3:**
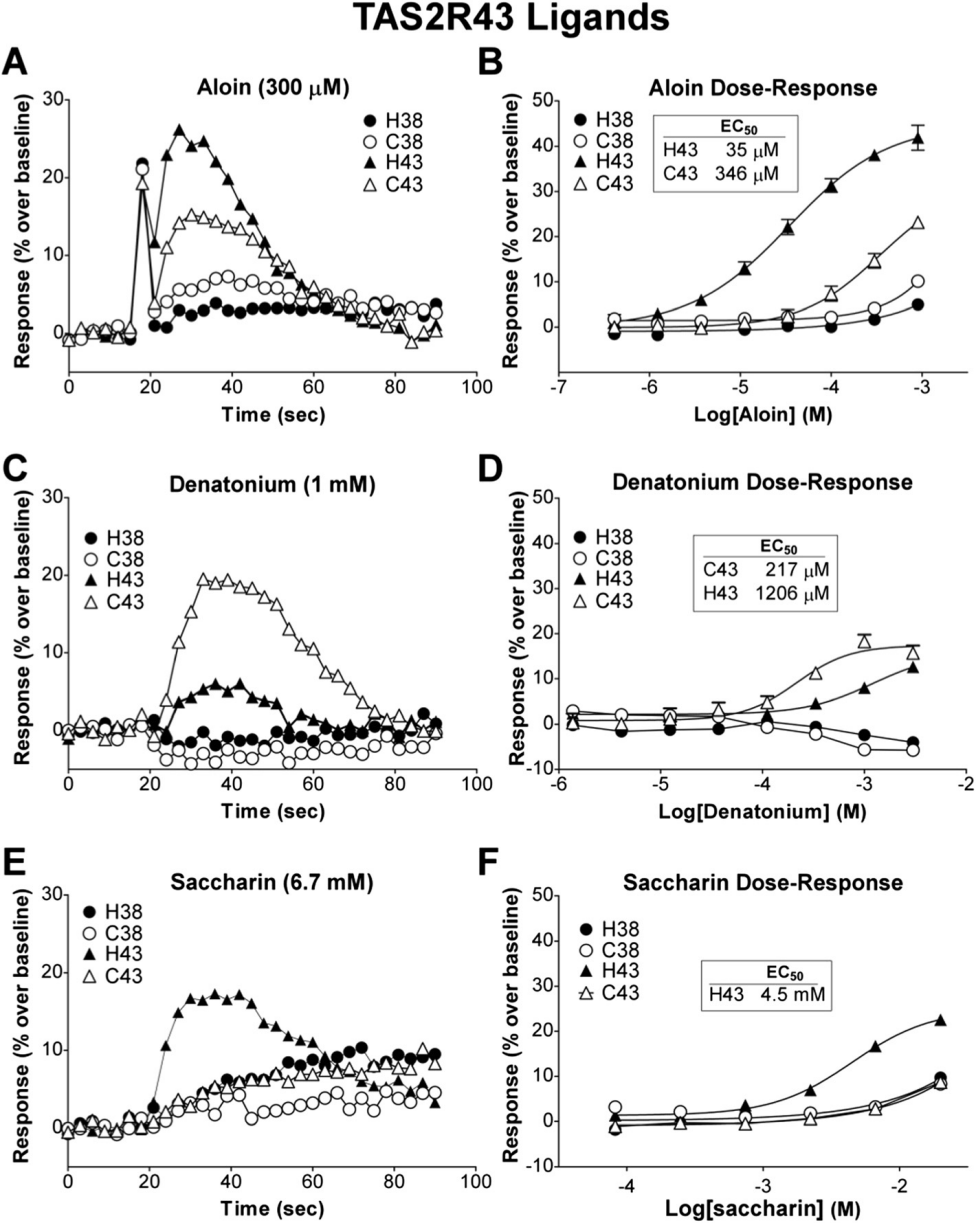
Human and cat TAS2R43 show different ligand responses and affinities. Cells were transiently transfected with Gα16-gust44 and the indicated receptors human TAS2R38 (H38) TAS2R43 (H43) cat Tas2r38 (C38) and Tas2r43 (C43). 22 hours post-transfection, calcium influx was measured in cells challenged with the ligands characteristic for human TAS2R43. (**a** & **b**) Both human and cat Tas2r43 responded to aloin in a dose-dependent manner (individual trace shown for 300 μM); however the cat Tas2r43 EC_50_ (346 μM) was roughly 10-fold higher than that of human TAS2R43 (35 μM). (**c** & **d**) While both human and cat TAS2R43 responded to denatonium, the cat receptor displayed greater sensitivity and a greater than 5-fold lower EC_50_ (217 μM) than human TAS2R43 (1206 μM). (**e**) Only human TAS2R43 responded to saccharin

Cat Tas2r43 responded to 300 μM aloin as did the human TAS2R43; however, the human receptor responded with a greater magnitude than the cat receptor (Fig. 3a). The calcium flux elicited by aloin for cells expressing cat Tas2r43 was significantly different than for cells expressing cat Tas2r38 (Additional file 4: Figure S4). In a dose–response analysis with aloin, the human receptor was about 10-fold more sensitive, with a lower EC_50_ than the cat receptor, 35 μM and 346 μM respectively (Fig. 3b). In contrast, the cat Tas2r43 receptor responded to 1 mM denatonium with a much higher magnitude of flux than the human TAS2R43 receptor (Fig. 3c). The dose–response analysis reflected this difference, with the cat Tas2r43 having an EC_50_ of 217 μM and human TAS2R43 an EC_50_ of 1206 μM (Fig. 3d).

Lastly, saccharin did not elicit a response from cells transfected with the cat Tas2r43 (Fig. 3e). At 6.7 mM saccharin, which elicited a response from human TAS2R43, cat Tas2r43 showed no calcium flux greater than the TAS2R38 receptors. The dose response confirmed that only the human TAS2R43 receptor responded specifically to saccharin (Fig. 3f).

The EC_50_ and Hill Slope for all the dose response curves are summarized in Table 1. Although the cat and human dose–response curves to aloin and denatonium did not reach saturation due to the limited solubility of the compounds, the EC_50_ values calculated from the available data can be considered as a lower limit for the EC_50_. In each case, this demonstrates a large difference in EC_50_ between the compared human and cat receptors. Furthermore, the human EC_50_ values are compared to published data and indicate similar value ranges. Thus our recombinant cellular system is producing responses similar to previous reports in the literature.

**Table 1:** **EC50 and Hill Slope values for human and cat TAS2R38 and TAS2R43**

		**TAS2R38 published**	**TAS2R38 human**	**Tas2r38 cat**	**TAS2R43 published**	**TAS2R43 human**	**Tas2r43 cat**
**PTC**	EC50	1.1 μM [19]	2.3 μM	47 μM	-	-	-
	Hill Slope	-	1.0	2.6	-	-	-
**PROP**	EC50	2.1 μM [19]	2.2 μM	-	-	-	-
	Hill Slope	-	1.59	-	-	-	-
**Aloin**	EC50	-	-	-	1.2 μM [20]	35 μM	346 μM
	Hill Slope	-	-	-	-	0.8	1.0
**Denatonium**	EC50	-	-	-	300 μM [5]	1206μM	217 μM
	Hill Slope	-	-	-	-	1.3	1.7
**Saccharin**	EC50	-	-	-	1.7 mM [44]	4.5	mM
	Hill Slope	-	-	-	-	1.5	-

Values were derived from the data shown graphed in Figs. 2 and 3

### Inhibition of cat Tas2r38 and Tas2r43 receptors by probenecid

Probenecid is an FDA-approved inhibitor of the organic anion transporter Multidrug Resistance Protein 1 (MRP1) and other organic anion transporters. In calcium flux studies, probenecid is commonly used to improve dye-loading by cells. Recently, probenecid was identified as an allosteric inhibitor of a subset of human TAS2R receptors [12], including human TAS2R38 and TAS2R43.

**Fig. 4:**
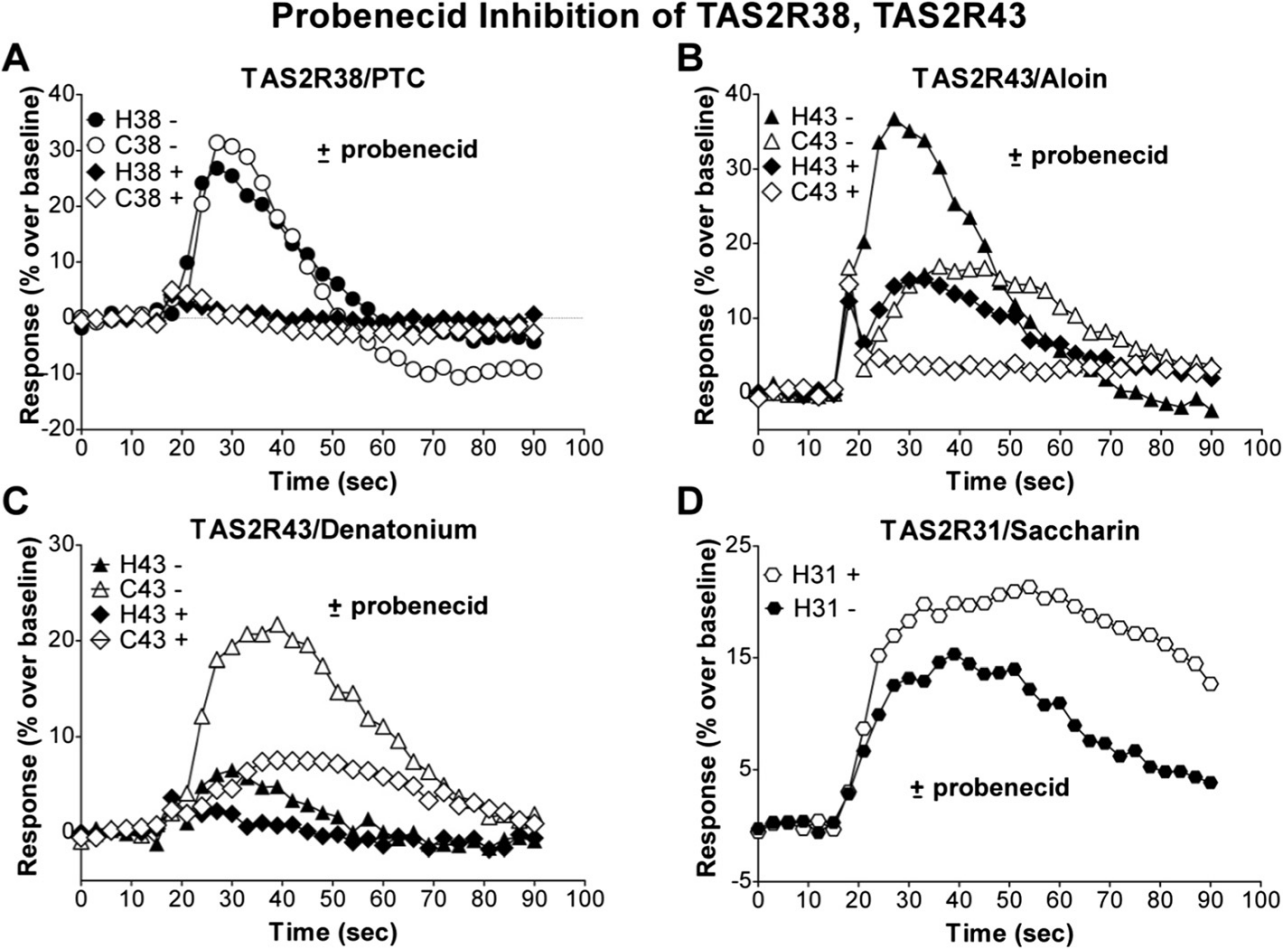
Probenecid inhibits both human and cat TAS2R38 and TAS2R43 response**.** To determine whether probenecid inhibited cat TAS2R38 and TAS2R43, the ability of both cat and human receptors to stimulate calcium flux was measured as above in the absence (−) of probenecid and after pre-incubation for one hour with probenecid at 1 mM (+). (**a**) Both human and cat TAS2R38 responses to PTC were inhibited completely by probenecid. Both human and cat TAS2R43 responses to aloin (**b**) and denatonium (**c**) were inhibited by probenecid. (**d**) The receptor-specific nature of probenecid inhibition was demonstrated by its stimulation of observed flux by human TAS2R31 response to saccharin [12]

We determined if probenecid could inhibit the response of cat bitter receptors to PTC, aloin or denatonium. For calcium flux experiments, transiently transfected cell lines expressing individual cat or human TAS2R receptors and Gα16-gust44 were pre-incubated for one hour with 1 mM probenecid prior to assays. In the presence of probenecid, stimulation with 33 μM PTC failed to induce a calcium flux in cells transfected with either the cat or the human TAS2R38 receptors (Fig. 4a).

Similarly, for both the cat and the human TAS2R43 receptors, pre-incubation with probenecid inhibited responses to 300 μM aloin and to 1 mM denatonium (Fig. 4b-c). The receptor-specific nature of probenecid inhibition was demonstrated by the probenecid stimulation of flux in response to saccharin in cells expressing human TAS2R31 (Fig. 4d), reflecting probenecid’s non-inhibition of TAS2R31 and its functional activity enabling a higher level of calcium-responsive dye to be retained within cells.

Thus, the inhibitory activity of probenecid appears to have been conserved for cat Tas2r38 and cat Tas2r43 despite the molecular and functional differences from their human counterparts.

## Conclusions

The prevailing hypothesis is that the ability to detect bitter tastants has evolved because of its utility in avoidance of toxic compounds often found in plants, yet obligate carnivores consume little to no plant material. Considering the technical and ethical difficulties in performing behavioral studies on domestic cats, in part due to a lack of feline toxicity data for many of the compounds, we rely on historical data which provides evidence that domestic cats are able to detect bitter compounds [26, 27] and reject those compounds [28, 31]. Domestic cats have the opportunity to encounter bitter compounds through numerous sources including eating plants, the plant constituents present in the gastrointestinal tract of prey, grains and flavors present in pet food as well as medicines [10, 32, 33]. A concern arises when a household cat is ill and either a specialized food or medicine is prescribed and refused. Pet owners and veterinarians desire strategies that will increase the consumption of nutrients and medicines when needed. Thus, to gain insight into cat bitter perception, we identified, functionally expressed, and deorphanized two cat genes predicted to encode bitter taste receptors, Tas2r38 and Tas2r43, where the role of the human orthologs in human bitter detection and perception is well understood [19, 20, 41, 44].

### Tas2r38

Several species within the order Carnivora show positive selection at the sensory gene *TAS2R38* [55]. This suggests that the detection of plant-derived thiourea-containing compounds has a positively-adaptive role for dogs, tigers and cats [55]. The human and cat TAS2R38 proteins are 67.6% identical, and as expected, cat Tas2r38 responded strongly to PTC, a cognate ligand of human TAS2R38. However, unlike human TAS2R38, the cat Tas2r38 was not responsive to PROP although it contains a combination of human TAS2R38 polymorphisms (Pro, Ala, Ile at residues 49, 262, and 295 respectively) associated with the ‘taster phenotype’ for PTC and PROP. Recent computational and functional studies of human TAS2R38 have identified additional residues involved in the recognition of these ligands [56, 57]. The majority of the human TAS2R38 amino acid residues which are involved in the direct binding of the ligand, receptor activation, or forming the binding pocket, are conserved in the cat sequence, for example, amino acids Trp99, Met100, Asn103, Phe197, and Ser259. A recent computational analysis predicted additional amino acid residues involved in either directly binding PROP and PTC or affecting the conformation of the residue-binding cavity. These residues were then tested functionally in a cellular assay [58]. Of the residues predicted and tested, three amino acids differed between the cat and the human TAS2R38 sequences: human Trp201, Ser260, and Phe264, which correspond to Ala200, Phe259, and Leu263 in cat Tas2r38. Trp201 is likely involved in receptor activation, Ser260 is involved in shaping the binding cavity and Phe264 is involved in binding the ligands [58]. Our data are not fully explained by the human receptor structure-function data as the cat Tas2r38 has a lower EC_50_ for PTC than the human receptor, and there was no response for PROP (Fig. 2b and d). No single mutation examined in prior human cellular assays mimicked the cat Tas2r38 response [58]; this suggests that multiple and non-overlapping residues are contributing to receptor activation by PTC and PROP. Chimeras between the human and cat receptor could provide useful insight into the ligand specificity within TAS2R38.

### Tas2r43

Human TAS2R43 and the predicted cat Tas2r43 are only 59% identical. To deorphanize cat Tas2r43, we tested its activation by 3 human agonists that characterize TAS2R43: denatonium, aloin and saccharin. The cat receptor was activated by aloin and denatonium only, an activation pattern distinct from that of human TAS2R43. Since only human TAS2R43 responds to both aloin and denatonium [5,54], we chose to operationally identify this receptor as cat Tas2r43. The response of human TAS2R43 to aloin has been characterized and the presence of Trp at polymorphic position 35 has been shown to be important for the sensitive human perception of aloin, and for human TAS2R43 activation in cellular assays [20]. Trp35 was present in the human TAS2R43 used in this study. Because Trp35 is located in the first intracellular loop this residue is likely involved in receptor activation and coupling to a G protein. Trp35 is conserved in the cat Tas2r43 receptor and its presence is consistent with cat Tas2r43 having a functional role in taste perception, as was suggested by the ability of cat Tas2r43 to respond to aloin and denatonium (Fig. 3). Although cats can detect saccharin and appear to avoid it [28, 59], our data suggests that this is not achieved through the activation of Tas2r43. Several other human receptors apart from TAS2R43 can respond to saccharin [5], thus it is likely an alternative cat bitter receptor is responsible for detection of this compound.

Comparative analysis alone of the human TAS2R38 and cat Tas2R38 sequences based on known ligand binding residues and polymorphisms did not predict the dichotomy of a receptor that responds to PTC and not PROP. Similarly it is nearly impossible to predict from the protein sequence alignment alone that the cat receptor would respond to denatonium and aloin but not saccharin. This underscores the importance of functional characterization of the cat bitter repertoire in understanding bitter taste perception in cats. Such studies can also provide further insight into the functional significance of specific amino acids and polymorphisms across species.

Research has shown that molecular diversity in the TAS2Rs of humans and other primates leads to functional differences in individuals’ bitter taste perception [40, 60]. The exposure to the specific flora of a geographic region is thought to be the major driving force of selection on TAS2Rs [21, 45]. In addition, bitter receptors are also expressed in the gastrointestinal and respiratory systems [61, 62] where they regulate metabolic and digestive processes and respiratory function, respectively. Thus it may be that felines, including domestic cats and other obligate carnivores, express extra-oral bitter receptors to regulate the digestive and respiratory systems as well as warn against bacterial or fungal contaminants as they are encountered.

In this study we have deorphanized two previously uncharacterized cat bitter receptors Tas2r38 and Tas2r43, and the data included in this study provide insights into the basic structure-function relationship of these receptors, showing that their functional characteristics are distinct from their human orthologs. This provides a basis for additional work to understand cat bitter perception that will also be valuable in formulating appetitive food for household cats as well as designing masking agents to enhance the acceptability of medications. Further investigation of the bitter receptor repertoire of large cats, such as lions and tigers, may reveal additional insight into the evolutionary history of this gene family.

## Methods

### Gene Identification and Comparisons

To identify cat Tas2r38 and Tas2r43, the equivalent human gene sequences were used (human TAS2R38 [GenBank:Gene ID 5726], human TAS2R43 [GenBank:Gene ID 25989]) as queries to perform a discontiguous megablast of the *Felis catus* whole-genome shotgun contigs database [46, 47]. Domestic cat contigs were downloaded from the NCBI database and the region of similarity identified in the BLAST search was then located. The sequences were screened for coding regions, “Start” and “Stop” codons, and translated protein length, as the typical bitter receptor is 300aa long. Confirmation of gene sequences was performed through cloning of the candidate gene. Cats used in this study live in an environment that simulates home situations, which includes ample opportunities to socialize with each other and the employees that staff the facility. Cats are group housed in compatible groups of 20–22 with daily outdoor access. Water is provided ad libitum and food is offered from 3 PM to 6 AM. This facility is audited annually by the USDA and meets or exceeds the standards set forth by the Animal Welfare Act. Experimental procedures were approved by the Institutional Animal Care and Use Committee of AFB International. The IACUC committee includes a non-affiliated member, a veterinarian and several non-scientist members as required by the USDA. The Institutional Official on the committee is the facility director, a Ph.D. animal nutritionist, with many years of experience in working with and managing facilities for companion animals to insure that the health and welfare of the residents are uncompromised. Genomic DNA was isolated from domestic cat saliva retrieved from individual cats by placing absorbent swabs in the back pouches of the mouth and following the Oragene∙ANIMAL kit as per the manufacturer’s recommendations (DNAgenotech, Kanata, Ontario, Canada). The candidate DNA fragment was amplified via PCR using Easy A High Fidelity PCR Cloning Enzyme (Agilent, Santa Clara CA), and custom primers (cat Tas2r38 Fwd 5’ – GAAGTCCTGGCTTGTAATGTA – 3’ reverse 5’ – CAAAACAAACTTGGGGAACTT – 3’, cat Tas2r43 Fwd 5’ – GCACAACCAGCGACATCAGACATT– 3’ reverse 5’ –CCCAGGCGCCCCAAAAGA– 3’), and the resulting PCR product was cloned into the pGEM-T Easy Vector (Promega, Madison WI). These clones were sequenced at the Core DNA Sequencing Facility at the University of Illinois, Champaign-Urbana. Analysis of sequences utilized the Lasergene suite of programs (DNAStar, Madison WI). Alignment of the human and cat sequences was performed in MegAlign (DNAStar, Madison WI) using the clustalW method.

### Expression Plasmids

Expression plasmids for human TAS2R16, TAS2R38, and TAS2R43 and chimeric G protein Gα16-gust44 were previously described [12]. Expression plasmids for cat Tas2r38 and Tas2r43 were created similarly, expressing full-length receptor under the control of a CMV promoter, with an N-terminal FLAG epitope tag, an SST3 signal sequence, and a C-terminal V5 epitope tag.

### Immunofluorescence assays

Each receptor construct was expressed in mammalian cells (ATCC CRL-11268) as described [12] and tested for surface expression by immunofluorescent antibody binding assays. Twenty-four hours post-transfection, cells were washed with Phosphate Buffered Saline (PBS) (HyClone), followed by addition of cell stripper (Cellgro). Suspended cells were fixed with paraformaldehyde at a final concentration of 4%. To detect surface expression of proteins, cells were incubated for one hour with anti-FLAG MAb M2 (1:500; Stratagene). To determine total (full-length) expression, cells were permeabilized using PBS with 0.1% saponin and incubated with an anti-V5 antibody (Invitrogen R960-25) for one hour. Primary antibody incubations were followed by a one-hour incubation with goat anti-mouse Cy3-conjugated secondary antibody (1:500; Jackson Laboratories). Secondary antibody fluorescence was measured from a minimum of 500 cells by flow cytometry on an Intellicyt HTFC screening system.

### Calcium Flux Assay

Wild-type TAS2R receptors were tested for function using a cell-based Ca^2+^ flux assay described previously [12], using the following compounds: probenecid, phenylthiourea (PTC), saccharin sodium salt hydrate (all from Sigma, St. Louis, MO), denatonium benzoate and aloin (Alfa Aesar, Ward Hill, MA), 6-n-propylthiouracil (PROP) (Selleck Chemicals) and C4-HSL (N-butyryl-L-homoserine; Cayman Chemical). Briefly, cells were transfected with the appropriate expression vector and a plasmid expressing a Gα16 chimera (Gα16-gust44), containing the last 44 amino acids of rat gustducin) in poly-lysine coated, black 384-well plates with clear bottoms (Costar). Cells were incubated for 22 hours at 37 °C then washed twice and loaded with a calcium indicator dye in HBSS containing 20 mM HEPES (Calcium 4 Assay kit, Molecular Devices), incubated for 1.5 hours, then moved to a Flexstation II-384 (Molecular Devices) set at 32 °C. Probenecid, a commonly used additive designed to improve dye-loading of cells, was not included for most incubations due to our previous demonstration of probenecid as a TAS2R16 inhibitor [12]. After a 10-minute temperature equilibration, ligand was injected (at t = 20 seconds) and fluorescence was measured for 60 seconds (reading every 3 seconds). Data sets were analyzed using Prism 5.0 software (GraphPad Software, Inc).

## Additional files

Additional file 1: Figure S1. Cats encode predicted protein orthologs to human bitter taste receptors 38 and 43. Additional file 2: Figure S2. Ligands used to deorphan cat Tas2r38 and Tas2r43. Additional file 3: Figure S3. C4-HSL activation of human TAS2R38 is statistically significant when compared to the human TAS2R43 response. Additional file 4: Figure S4. Aloin activation of cat Tas2r43 is statistically significant when compared to the cat Tas2r38 response.
