# Supplementary material for: A functional comparison of the domestic cat bitter receptors Tas2r38 and Tas2r43 with their human orthologs
Source: BMC Neurosci. 2015 Jun 3;16:33. doi: 10.1186/s12868-015-0170-6 (PMC4453034; doi:10.1186/s12868-015-0170-6)
Supplement: Additional file 1: Figure S1. — Cats encode predicted protein orthologs to human bitter taste receptors 38 and 43. [file 12868_2015_170_MOESM1_ESM.pdf]

Supplementary
Figure 1

A.

```
                  10         20         30         40         50
cT2r38    MLALTPVITV SYEVKSAFLF LSILEFTVGV LANAFIFLVN FWDVVRKQPL   50
hT2R38    MLTLTRIRTV SYEVRSTFLF ISVLEFAVGF LTNAFVFLVN FWDVVKRQAL   50

                  60         70         80         90        100
cT2r38    SNCDLILLSL SLTRLFLHGL LFLDALQLTY FQRMKDPLSL SYQTIIMLWM  100
hT2R38    SNSDCVLLCL SISRLFLHGL LFLSAIQLTH FQKLSEPLNH SYQAIIMLWM  100

                 110        120        130        140        150
cT2r38    ITNQVGLWLT TCLSLLYCSK IARFSHTLLH CVASWVSRKV PQMLLGAMLF  150
hT2R38    IANQANLWLA ACLSLLYCSK LIRFSHTFLI CLASWVSRKI SQMLLGIILC  150

                 160        170        180        190        200
cT2r38    SCICTAICLG DFFSRSGFTF TTMLFVNN-T EFNLQIAKLS FYHSFIFCTL  199
hT2R38    SCICTVLCVW CFFSRPHFTV TTVLFMNNNT RLNWQIKDLN LFYSFLFCYL  200

                 210        220        230        240        250
cT2r38    ASIPSLLFFL ISSGVLIVSL GRHMRTMRAK TKDSHDPSLE AHIKALRSLV  249
hT2R38    WSVPPFLLFL VSSGMLTVSL GRHMRTMKVY TRNSRDPSLE AHIKALKSLV  250

                 260        270        280        290        300
cT2r38    SFLCLYVVSF CAALVSVPLL MLWHNKIGVM ICVGILAACP SIHAAILISG  299
hT2R38    SFFCFFVISS CVAFISVPLL ILWRDKIGVM VCVGIMAACP SGHAAILISG  300

                 310        320        330
cT2r38    NAKLRRAVET ILLWVQNSLK IGADHKADAR TPGLC.                 335
hT2R38    NAKLRRAVMT ILLWAQSSLK VRADHKADSR T--LC                  333
```

B.

```
                  10         20         30         40         50
cT2r43    MVTALPSIFS IVVIIEFLLG NFANGFIALV NFIDWTKRQK ISSVDHILTA   50
hT2R43    MITFLPIIFS SLVVVTFVIG NFANGFIALV NSIEWFKRQK ISFADQILTA   50

                  60         70         80         90        100
cT2r43    LAVSRIGLLW VILINWYATL FSPDFYSLEV RIIFQTAWTV SNHFSIWLAT  100
hT2R43    LAVSRVGLLW VLLLNWYSTV LNPAFNSVEV RTTAYNIWAV INHFSNWLAT  100

                 110        120        130        140        150
cT2r43    SLSIFYLFKI ANFSSLIFLR LKWRVKSIVL VILLGSLFFL VCHVVAVSVC  150
hT2R43    TLSIFYLLKI ANFSNFIFLH LKRRVKSVIL VMLLGPLLFL ACHLFVINMN  150

                 160        170        180        190        200
cT2r43    EKVQTDVYEG NGTRKTKLRD ILQLSNMTIF TLANFIPFGM SLTSFVLLIF  200
hT2R43    EIVRTKEFEG NMTWKIKLKS AMYFSNMTVT MVANLVPFTL TLLSFMLLIC  200

                 210        220        230        240        250
cT2r43    SLWKHLKRMQ LCDKGSQDPS TKVHIRAMQT VVSFLLFFAG YFFTLTITIW  250
hT2R43    SLCKHLKKMQ LHGKGSQDPS TKVHIKALQT VISFLLLCAI YFLSIMISVW  250

                 260        270        280        290        300
cT2r43    SSNWPQNEFG FLLCQVIGIL YPSIHSLMLI RGNKKLRQAF LSFLWQLKC.  300
hT2R43    SFGSLENKPV FMFCKAIRFS YPSIHPFILI WGNKKLKQTF LSVFWQMRYW  300

cT2r43                                                         300
hT2R43    VKGEKTSSP                                            309
```

Supplementary
Figure 1

Supplementary Figure 1. Cats encode predicted protein orthologs to human bitter taste receptors 38 and 43. Sequences of human bitter taste receptors TAS2R38 and TAS2R43 were used to identify cat genes that were predicted to encode orthologous proteins. (**A**) Alignment of predicted cat Tas2r38 (cT2r38) with human TAS2R38 (hT2R38). (**B**) Alignment of predicted cat Tas2r43 (cT2r43) with human TAS2R43 (hT2R43).
