# Supplementary material for: A functional comparison of the domestic cat bitter receptors Tas2r38 and Tas2r43 with their human orthologs
Source: BMC Neurosci. 2015 Jun 3;16:33. doi: 10.1186/s12868-015-0170-6 (PMC4453034; doi:10.1186/s12868-015-0170-6)
Supplement: Additional file 2: Figure S2. — Ligands used to deorphan cat Tas2r38 and Tas2r43. [file 12868_2015_170_MOESM2_ESM.pdf]

Supplementary
Figure 2

| Ligand | Structure |
|---|---|
| PTC | 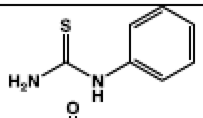 |
| PROP | 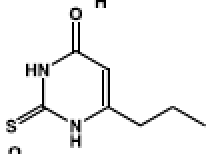 |
| C4-HSL | 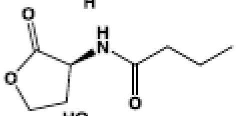 |
| Aloin | 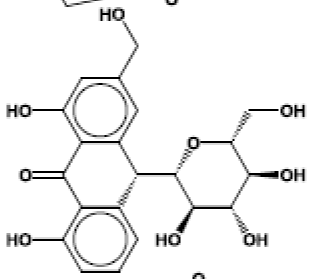 |
| Saccharin | 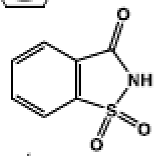 |
| Denatonium | 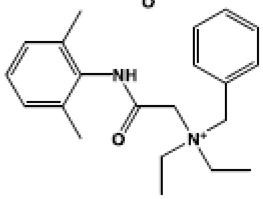 |
| Probenecid | 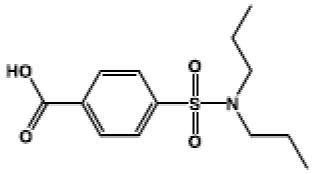 |

**Figure 2. Ligands used to deorphan cat Tas2r38 and Tas2r43.** The receptors were tested for their responses to known ligands for human TAS2R38 (phenylthiocarbamide (PTC ), 6-n-propylthiouracil (PROP), and C4-HSL (N-butyryl-L-homoserine lactone), TAS2R43 (aloin, denatonium, and saccharin), and for effects of probenecid, an inhibitor of some TAS2R receptors [12].
