# Supplementary material for: A functional comparison of the domestic cat bitter receptors Tas2r38 and Tas2r43 with their human orthologs
Source: BMC Neurosci. 2015 Jun 3;16:33. doi: 10.1186/s12868-015-0170-6 (PMC4453034; doi:10.1186/s12868-015-0170-6)
Supplement: Additional file 3: Figure S3. — C4-HSL activation of human TAS2R38 is statistically significant when compared to the human TAS2R43 response. [file 12868_2015_170_MOESM3_ESM.pdf]

Supplementary
Figure 3

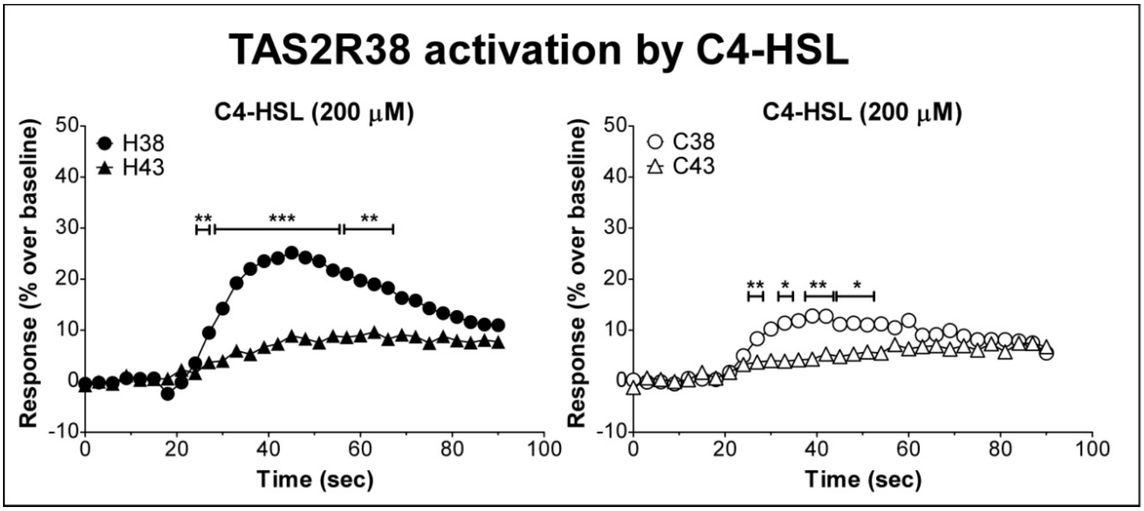

Supplementary Figure 3. C4-HSL activation of human TAS2R38 is statistically significant when compared to the human TAS2R43 response. Each point represents the average of 4 replicate measurements. For each time point the 4 baseline-corrected values for TAS2R38 and TAS2R43 were compared by unpaired, two tailed t-test. Significant differences are indicated by bars, with indicated P values (* < 0.05, ** < 0.01, *** < 0.001).
