# Supplementary material for: A functional comparison of the domestic cat bitter receptors Tas2r38 and Tas2r43 with their human orthologs
Source: BMC Neurosci. 2015 Jun 3;16:33. doi: 10.1186/s12868-015-0170-6 (PMC4453034; doi:10.1186/s12868-015-0170-6)
Supplement: Additional file 4: Figure S4. — Aloin activation of cat Tas2r43 is statistically significant when compared to the cat Tas2r38 response. [file 12868_2015_170_MOESM4_ESM.pdf]

Supplementary
Figure 4

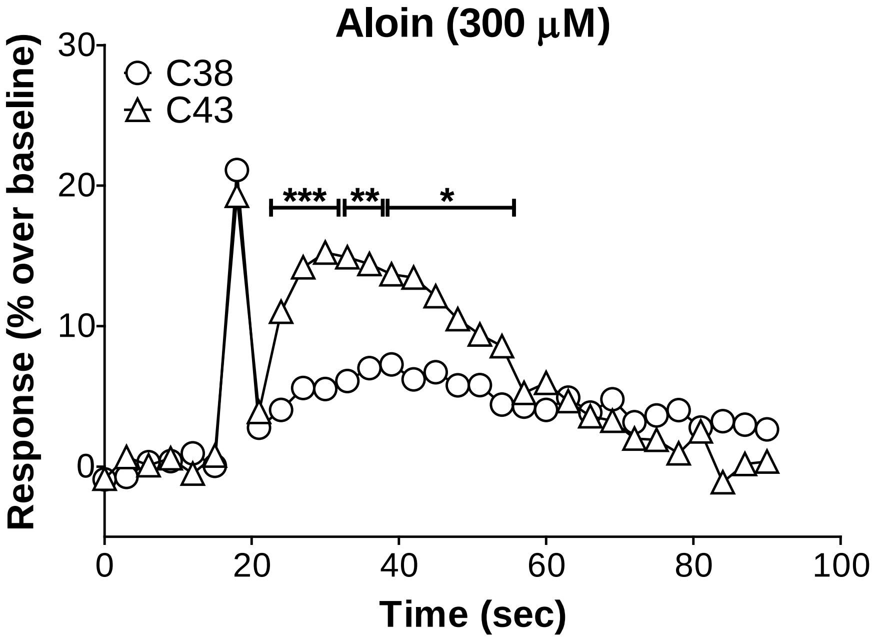

Supplementary Figure 4. Aloin activation of cat Tas2r43 is statistically significant when compared to the cat Tas2r38 response. Each point represents the average of 4 measurements. For each time point the 4 baseline-corrected values for TAS2R38 and TAS2R43 were compared by unpaired, two tailed t-test. Significant differences are indicated by bars, with indicated P values (* < 0.05, ** < 0.01, *** < 0.001).
